# Recent Development of Nanomaterials for Chemical Engineering

**DOI:** 10.3390/nano14050456

**Published:** 2024-03-01

**Authors:** Meiwen Cao

**Affiliations:** State Key Laboratory of Heavy Oil Processing, Department of Biological and Energy Chemical Engineering, College of Chemistry and Chemical Engineering, China University of Petroleum (East China), Qingdao 266580, China; mwcao@upc.edu.cn

There has been an explosive growth in research on nanomaterials since the late 1980s and early 1990s. So far, massive amounts of nanomaterials have been developed and their preparation and characterization methods are relatively comprehensive and well-established [1,2]. The current most important issue is the engineering of nanomaterials in real-world applications to promote economic and social development. Based on the booming development of nanomaterials in the field of chemical engineering, we have launched two Special Issues, that is, Nanomaterials for Chemical Engineering (Volume I, 2022) and Nanomaterials for Chemical Engineering (Volume II, 2023). Detailed information on Volume I can be found via the following web link: https://www.mdpi.com/journal/nanomaterials/special_issues/nano_chemical_engineering. In the latest Special Issue (Volume II), we collected 15 original research articles and 1 comprehensive review paper written by excellent scientists from relevant fields covering the topics of nanomaterial synthesis and characterization, the engineering of nanomaterials into composites with novel properties, the development of functional nanomaterials for applications in catalysis and pollutant treatment, the evaluation of the durability and environmental friendliness of nanomaterials, etc. This Special Issue also follows closely the forefront of global scientific research and includes studies on the application of machine learning in the field of nanomaterials for chemical engineering. In what follows, I will give a brief introduction of the studies presented in this Special Issue.

The design and optimization of nanomaterial synthesis as well as performance control are eternal themes in the development of nanotechnology [3]. The rapid advancement of material preparation technology and the actual needs of specific nanomaterials with desired properties both drive the development of new synthesis methods to produce novel nanostructures. In this Special Issue, Mares-Briones et al. presented the chemical synthesis of AgPt nanoalloys by the polyol method, which used polyvinylpyrrolidone (PVP) as a surfactant in a heterogeneous nucleation approach. This study modulated well the composition, the size and morphology, the catalytic activity as well as the stability and long-term durability of the AgPt nanoparticles, providing attractive catalyst candidates for cost-effective ethanol oxidation. Abitaev et al. reported the controlled synthesis of ZnO nanoparticles via the chemical bath deposition (CBD) method. The kinetics of ZnO formation was controlled by using a methanolic precursor solution containing the organic additive polyvinylpyrrolidone (PVP) as the stabilizing and structuring agent. They also gave a nearly quantitative description of both the nucleation and growth period using the two-step Finke–Watzky model with slow, continuous nucleation followed by autocatalytic growth. This study provides valuable insights into the controlled synthesis of ZnO nanoparticles. Quiñones-Gurrola et al. carried out a systematic study of preparing perovskite-type fine particles in the binary system of SrZrO_3_–SrTiO_3_ with the hydrothermal method. They optimized the synthesis parameters such as temperature, the precursors and their stoichiometry, and the reaction time. The size and structural characteristics as well as the crystalline nature of the nanoparticles were also systematically investigated. This study demonstrates that soft chemistry hydrothermal processing can be employed for synthesizing inorganic perovskite binary systems for potential industrial applications. Moreover, Altena et al. reported in their work the attempt to synthesize VBi_2_Te_4_ by molecular beam epitaxy (MBE) as well as the detailed characterization of the material by various techniques. Noting that VBi_2_Te_4_ is an intrinsic magnetic topological insulator (IMTI) that has been theoretically predicted but has a lack of experimental evidence supporting it so far, this study filled in this gap by providing experimental results for its material synthesis.

How to engineer a specific nanomaterial into practical applications, that is, the development of applying techniques, is crucial. In this Special Issue, Lazauskas et al. investigated the hydrophilic surface modification of SiO_x_ containing amorphous hydrogenated carbon nanocomposite films (DLC:SiO_x_) by using atmospheric oxygen plasma treatment. They showed that atmospheric oxygen plasma treatment can effectively modify the wetting property of the DLC:SiOx film, which can ensure practical applications such as biocompatible coatings for medical purposes, anti-fog coatings for optical components, and protective coatings to prevent corrosion and wear. Mills et al. formulated catalytic zero-valent iron/palladium (Fe/Pd) with stimuli-responsive poly-N-isopropylacrylamide (PNIPAm) and poly-methyl methacrylate (PMMA) to functionalize hollow fiber membranes, producing functional composite membranes for the reductive degradation of organic pollutants. By using trichloroethylene and methyl orange as model compounds for degradation, they proved that temperature-responsive domains and catalyst incorporation in the composited membrane provide significant advantages for toxic organic decontamination, which are promising for the efficient treatment of high-volume contaminated water. In another study included in the Special Issue, Chiang et al. synthesized nano-branched iridium nanodendrites (Ir NDs) on an antimony tin oxide (ATO) support (Ir NDs/ATO) by a surfactant-mediated method. They investigated the effect of ATO support and evaluated the electrocatalytic activity and durability of the material during an oxygen evolution reaction (OER). The authors’ detailed characterization of the structural properties and catalytic performance of the Ir NDs provides solid support for the practical application of this material.

By having an extremely small size and high specific surface area, nanomaterials have more active sites for improving the reaction efficiency in catalytic processes [4,5,6]. This Special Issue includes several papers on the chemical engineering applications of nanocatalytic materials, providing comprehensive explorations on materials preparation, physical–chemical characterization, and catalytic performance evaluation. The hybridization of nanomaterials with different structures and properties to produce composite materials is an important approach for engineering nanomaterials in various applications [7,8,9]. For example, two-dimensional (2D) graphene/graphene oxide (GO) and noble metal nanoparticles are two types of nanomaterials with distinct structural characteristics and physicochemical properties. The hybridization of them into a single composite material can produce new materials with exceptional performance due to the synergism of the specific properties of each material. In this Special Issue, Iordache et al. present a comprehensive review on the most used and up-to-date methods for the synthesis and characterization of graphene/noble metal (Pt, Ag, Pd and Au) nanocomposites as well as their application as new catalysts in fuel cell and renewable technology. This paper discusses well the preparation methods, the type and amount of noble metal, the nature of the graphene support material, the type of dopant and the metal–support relationship in the synthesis of graphene/noble metal nanocomposites. The electrocatalytic activity and electrochemical stability of the nanocomposites as well as the techniques of applying the nanocomposites as catalysts are also discussed. In another research paper, Marinoiu et al. developed a microwave-based single-step synthesis method for nitrogen-doped graphene oxide preparation, which is simple, faster, scalable, and economical. By evaluating and comparing the physical and chemical properties of various materials obtained from different precursors, they concluded that ammonia allowed for a higher nitrogen doping concentration by utilizing the high vapor pressure to facilitate the functionalization reaction with graphene oxide. This study contributes well to the synthesis methodology and engineering of nitrogen-doped graphene as a promising metal-free catalyst for the oxygen reduction reaction (ORR). Such catalysts show good electrocatalytic activity and long-term operation stability, and are excellent for practical ORR application in proton exchange membrane fuel cells (PEMFCs).

By having a high specific surface area and porosity as well as excellent catalytic capability, nanomaterials also find widespread applications in pollution treatment, that is, being used as adsorbents for the adsorptive removal of harmful substances from the environment or catalytic transformation of toxic species into nontoxic ones [10,11,12]. In this Special Issue, La Greca et al. synthesized composite catalysts of Ag/MnO_x_ and Ag/CeMnO_x_ by using a combination of the citrate sol–gel method for support synthesis and incipient wetness impregnation with [Ag(NH_3_)_2_]NO_3_ aqueous solution to deposit the active component. They systematically investigated the physical–chemical properties of the as-prepared catalysts by various characterization techniques and then studied the selective catalytic NO_x_ reduction with propylene. This work presents well how to develop the hybridized nanocatalysts for NO reduction applications. Saldaña-Robles et al. presented in their paper the synthesis of amine and ferrihydrite functionalized graphene oxide for the removal of fluoride from water. The comprehensive characterization of the synthesized materials was performed by using various techniques. This study is a good example of engineering composite nanomaterials for application in pollution treatment. In another work, Lu et al. [13] synthesized a novel organic–inorganic hybrid material of IIGK@MnO_2_ via an environmentally friendly and simple method. They used short peptide self-assembled nanostructures as a template to assist with the formation of a fibrous IIGK@MnO_2_ nanocomposite with a large specific surface area and negative charges, which can be used as an effective adsorbent for the removal of strontium ions (Sr^2+^) from aqueous solution. This study shed light on the construction of organic–inorganic hybrid adsorbents with multiple active adsorption sites and a high adsorption efficiency for adsorbing radioactive ions from wastewater.

Nanomaterials can be composited into other materials as fillers to enhance the mechanical properties such as strength, hardness, toughness, wear resistance, etc. [8] For example, two-dimensional hexagonal boron nitride (hBN) has a stable structure and outstanding properties such as mechanical strength, thermal conductivity, electrical insulation, and lubricant behavior. However, how to utilize hBN in practical applications is a significant challenge. Magaletti et al. used hBN as a filler material to partially replace silica in elastomer composites so as to improve their rheological and mechanical properties. They found that hBN as a substitute for 30% of the silica can greatly improve the material’s property, which leads to a lower Payne effect, a higher dynamic rigidity, and an increase in E′ with similar/lower hysteresis. This study paves the way for substantial improvements in the important properties of silica-based composites for tire compounds, which can be used to reduce rolling resistance and lessen environmental impacts.

For the practical application of a specific nanomaterial in chemical engineering, it is important to evaluate the performance, stability, safety, and environmental sustainability of the materials in advance [14,15]. However, such studies are relatively scarce at the current stage. In this Special Issue, Barjoveanu et al. presented in their paper how a life cycle assessment can be used to evaluate the eco-design options for early-stage material development and engineering while allowing for the environmental sustainability of novel materials. They focused on a comparison of the technical and environmental performance of two types of synthesis strategies (the classic layer-by-layer strategy and the one-pot coacervate deposition strategy) for PEI-coated silica particles (organic/inorganic composites), which were tested for Cd^2+^ ion removal from aqueous solution. This study proves the usefulness of life cycle assessments and scenario analyses as environmental support tools for engineering nanomaterials into practical applications because they can highlight environmental hotspots and point out the environmental improvement possibilities from the very early stages of material development. Pacella et al. adopted a multi-analytical approach to investigate the dissolution process and surface characterization of amosite fibers following interaction with a mimicked Gamble’s solution for a long period. This study highlighted the incongruent behavior of the amosite fiber dissolution and observed a preferential release of Mg and Ca from the amphibole structure as well the oxidation of Fe. By probing into the most important toxicity parameters, that is, the biodurability (i.e., the resistance to dissolution), this study helps us to understand the mechanisms of long-term toxicity, which is crucial for practical applications of UICC amosite.

Machine learning technology provides new ideas and methods for the research and engineering application of nanomaterials, helping to accelerate material innovation, optimize process design, and promote the practical application of nanomaterials. Wang et al. presented a good attempt of machine-learning-assisted synthesis of nanomaterials. They constructed a machine learning Gaussian regression model to assist with the preparation of molybdenum disulfide (MoS_2_) semiconductors with the CVD method and to explore the growth mechanism. The optimal model can predict the size of MoS_2_ synthesized under 185,900 experimental conditions in the simulation dataset, which enabled the selection of the optimal range for the synthesis of large-area MoS_2_. This study verifies that machine learning is a powerful tool for the development of nanomaterials in chemical engineering.

In short, this Special Issue achieves great success by presenting all of the above high-quality original research papers and comprehensive review papers. We give our sincere thanks to the excellent scholars that have made contributions to this Special Issue. Inspired by the previous two successful Special Issue volumes, we are now launching the third volume of the Special Issue, that is, “Nanomaterials for Chemical Engineering III”. We welcome even more excellent scholars to submit their original research or review papers on nanomaterials for chemical engineering to the Special Issue. Besides the topics that were covered by the previous two volumes (e.g., nanomaterials synthesis and characterization, catalytic nanomaterials, environmental protection and pollution control, biomedical applications, computational modeling studies, as well as utilization in devices and practical applications), we will welcome contributions from new fields, including, but not limited to, the following topics:

Green chemical engineering: Utilizing the special properties and effects of nanomaterials to develop green and sustainable chemical reactions and processes [16]. For example, the design and application of novel nanocatalysts will help to improve reaction selectivity and efficiency, reduce energy consumption and waste generation.

Energy applications: Nanomaterials have enormous potential in the energy field [17]. For example, nanomaterials can be used as electrodes and electrolytes for efficient energy storage devices, improving the performance of batteries and supercapacitors [18]. Nano photocatalysts can be used for photocatalytic water splitting and hydrogen production, etc.

The sustainability of nanomaterials: The preparation and application of nanomaterials need to consider their impact on the environment. The development of more sustainable and environmentally friendly preparation methods, as well as the recovery and recycling of nanomaterials, are key issues [19].

Moreover, we greatly encourage contributions from artificial intelligence and machine learning. These fast-developing artificial intelligence (AI) technologies provide great opportunities for the research and application of nanomaterials in the field of chemical engineering [20,21]. However, the design and optimization of machine learning algorithms also need to fully consider the special properties of nanomaterials themselves so as to obtain accurate and reliable results [22]. We believe that machine learning will provide many potential advantages and innovations to the engineering application of nanomaterials, including material design and prediction, reaction control and optimization, the analysis and interpretation of massive experimental data, the improvement of experimental efficiency, and so on.
